# Posterior Cerebral Circulation Insufficiency Due to Internal Carotid Artery Stenosis With an Atypical Vertebral Artery Originating From the Internal Carotid Artery Territory

**DOI:** 10.7759/cureus.75972

**Published:** 2024-12-18

**Authors:** Mahetab Shehata, Leen Foudeh, Marina Khoury, Matthew Armon, Wissam Al-Jundi

**Affiliations:** 1 Vascular Surgery, Royal Free Hospital, London, GBR; 2 Vascular Surgery, Norfolk and Norwich University Hospitals NHS Foundation Trust, Norwich, GBR

**Keywords:** aberrant vertebral artery, abnormal cerebral anatomy, carotid artery surgery, carotid stenosis, cerebral circulation

## Abstract

Aberrant anatomical variation of the vertebral artery (VA) from an internal carotid artery (ICA) is considered a rare finding. The incidence of this phenomenon can lead to patients suffering from posterior circulation neurological deficit if the ICA becomes significantly diseased.

VA atypical anatomical origin is considered one of the rare pathologies, not only precipitating neurovascular incidents but equally leading to severe difficulty in VA dissection and surgical exposure, especially in carotid artery procedures. The "vertebral arteria lusoria," despite its rare incidence, ought to be considered in cases undergoing upper gastrointestinal surgeries; thus, not knowing this pathology may lead to life-threatening incidents. Furthermore, double origins of VA could be noticed in the pre-operative assessment of cases with extracranial pathology. Strokes in the posterior cerebral vascular territory, which are vertebral-basilar arteries and spinal cord perfusion deficiency, are frequently mentioned impediments to VA injury in patients with normal or aberrant VA origins. Consequently, dominant atypical VA, in absentia, atresia of the artery, completely thrombosed contralateral VA, and failure to assess the vertebrobasilar perfusion are solid triggering signs to preserve subclavian artery and atypical VA perfusion.

We report a rare case of a 76-year-old female who presented with intermittent dizziness, left facial numbness, and left arm paresthesia. On investigation with computed tomographic angiography (CTA), she was incidentally found to have an aberrant anatomy of a dominant right VA arising from her right ICA and severe stenosis in the right ICA origin of 70% significant stenosis. Patients with posterior circulation symptoms and ICA stenosis may have aberrant anatomy. High-quality imaging in the form of CTA is essential prior to deciding whether and what surgery is indicated.

## Introduction

Vertebral arteries supply the posterior cerebral circulation; this artery originates from the subclavian artery, precisely from the upper border of the first segment. Several anatomical variations of vertebral artery (VA) origin have been reported in the literature. It might originate from the aorta, the common carotid artery, or the brachiocephalic artery or have a dual origin. Still, few cases of VA originating from the internal carotid artery (ICA) have been documented [1]. Multiple aberrant origins of bilateral vertebral arteries have been documented. The commonest aberrancy is the left VA originating from the aortic arch between the left common carotid artery and the left subclavian artery; on the contrary, the right VA originating from the aortic arch is very rare [2]. A VA with significant stenosis can result in posterior circulation neurological events with non-specific symptoms and signs such as nausea, vomiting, dizziness, and major motor and sensory deficits. Most case reports mentioned an association with an aberrant subclavian artery, specifically on the right side. Although it is not associated with any signs or clinical symptoms, awareness of the exact position of the atypical VA should be assessed when surgical options and percutaneous interventions are considered, including the arch of the aortic and descending thoracic aorta. In this case report, we report an extremely rare case of aberrant origin of the right VA from the ICA with significant 70% stenosis in a patient presenting with symptoms consistent with anterior and posterior right hemispheric stroke.

## Case presentation

**Figure 1:**
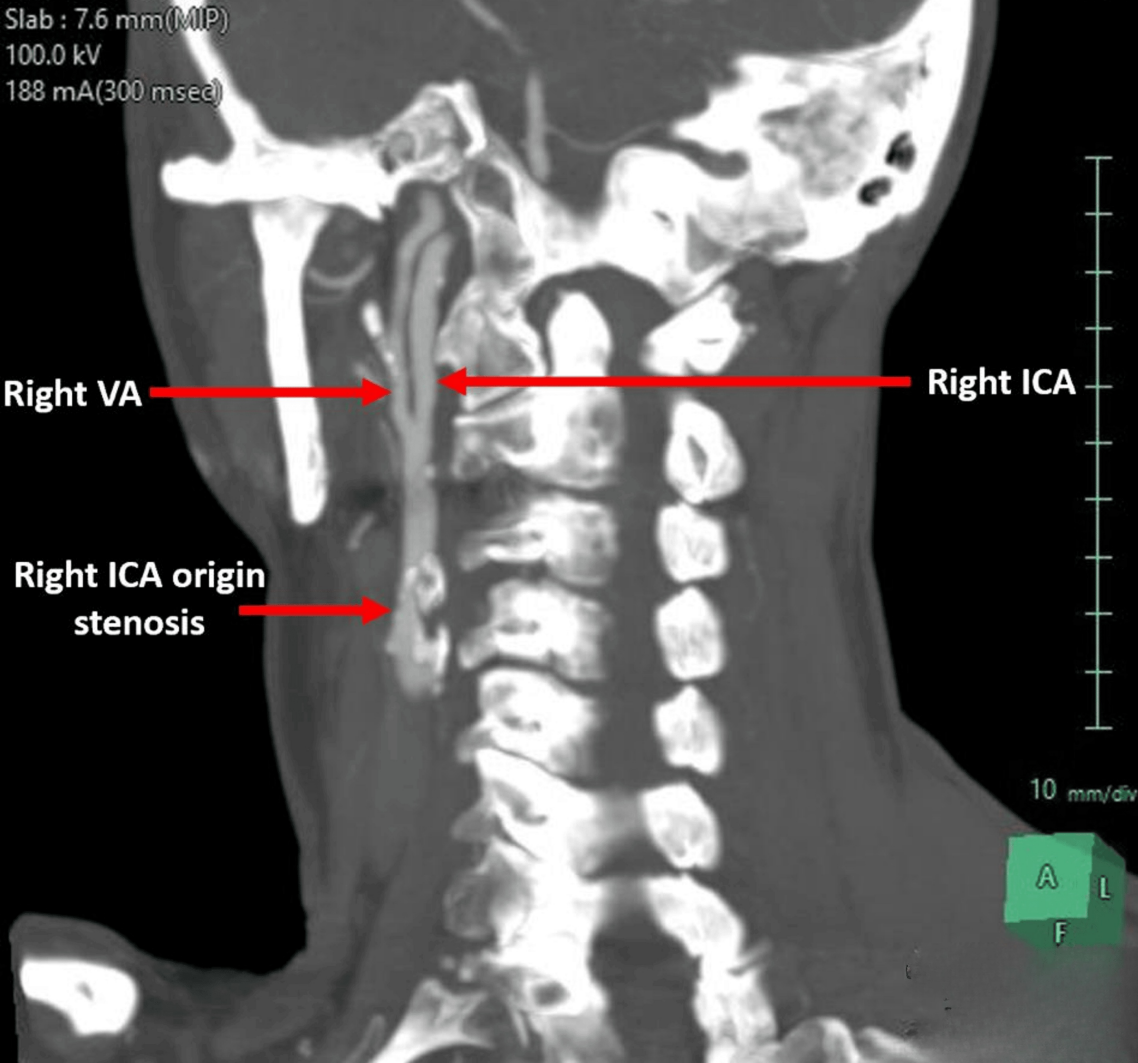
CTA demonstrating the main right VA arising from the right distal ICA with stenosis in the ICA CTA: computed tomographic angiography, VA: vertebral artery, ICA: internal carotid artery

A 76-year-old female was admitted complaining of intermittent dizziness that lasted for longer than a week, left facial numbness for three days, and a sudden onset of left arm paresthesia, which is a transient ischemic attack. On examination, she had no focal neurological deficits, her pulse was regular, and she had right carotid bruit on auscultation. She had a past medical history of scleroderma, Raynaud’s disease, recurrent transient ischemic attacks, and vertigo. She is known to be fit and active for her age, lives with her partner, and does her activities alone. She has no previous history of myocardial infarction or angina, is a non-smoker, and has normal kidney function. She had carotid duplex as the first investigation, showing significant stenosis of right ICA 70% with chronic large plaque, but couldn't comment on the VA or any abnormal anatomy. Further investigations with computed tomographic angiography (CTA) of the aortic arch and both carotids (Figure 1) demonstrated a very unusual anatomy of a dominant right VA arising from her right distal ICA and a calcified atheromatous plaque in the right ICA origin, causing 70% stenosis.

**Figure 2:**
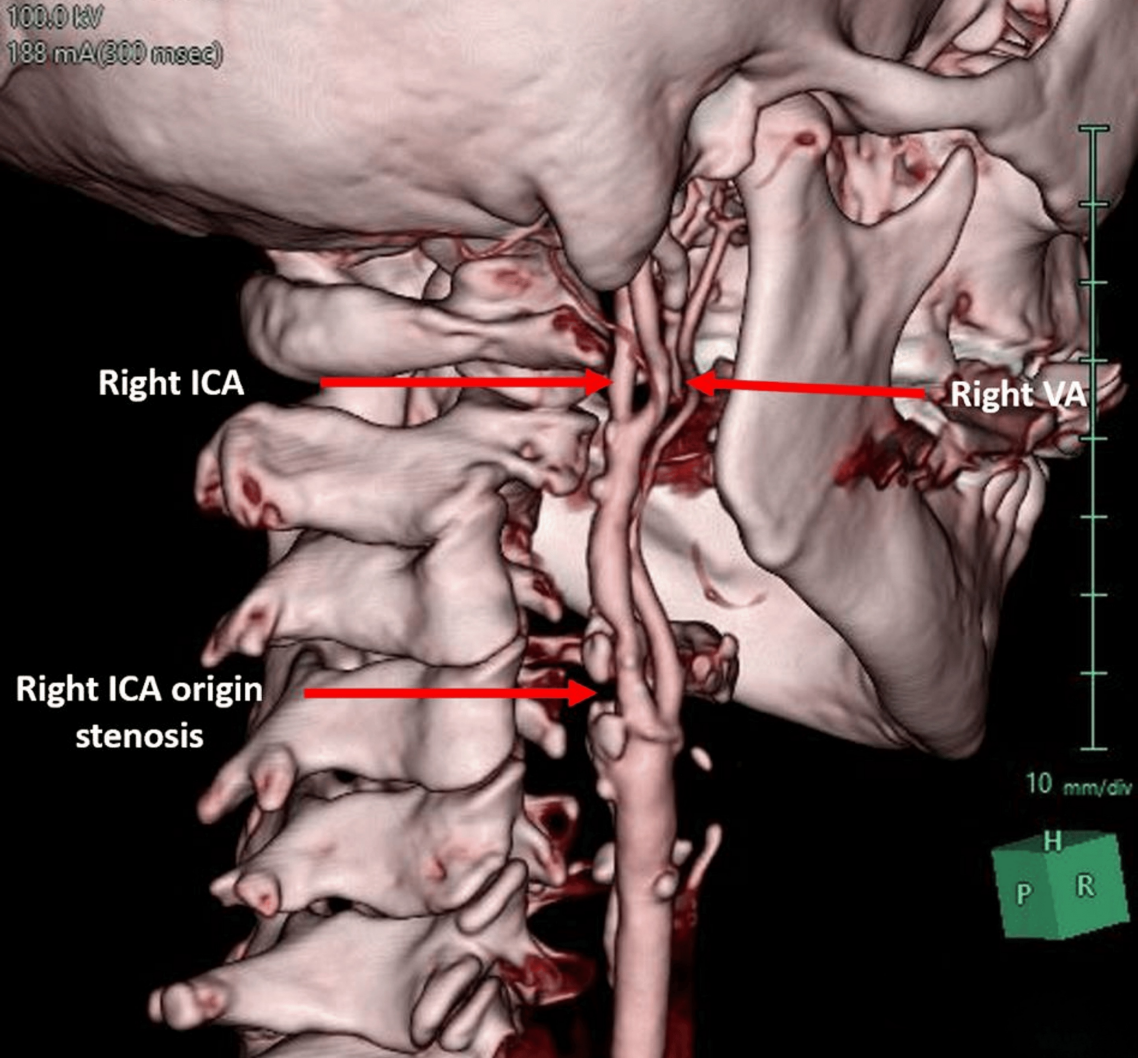
3D reconstruction of carotid vessels showing significant 70% ICA stenosis and aberrant VA 3D: three dimensional, ICA: internal carotid artery, VA: vertebral artery

A duplex ultrasound scan confirmed a 25-mm-long calcified atheroma, and it was estimated that the degree of proximal ICA stenosis was 70% as well. Her left common and internal carotid arteries were patent with normal color filling and Doppler signals, and her left VA followed a predictable anatomical origin from the left SCA. CT and MRI heads were unremarkable (Figure 2).

The patient underwent a right carotid endarterectomy with a patch repair. There were no perioperative complications; she made an uneventful recovery and was discharged on 75 mg clopidogrel life-long. Six weeks post-operatively, both her anterior and posterior circulation symptoms had resolved.

## Discussion

The posterior circulation of the brain consists of two vertebral arteries, which unite together to form the basilar artery. The former artery consists of four segments. The proximal three segments are considered the extracranial part, while the distal fourth part is the intracranial part. V1 starts from the superior aspect of the subclavian artery and ascends to the transverse foramen of the sixth cervical vertebra; V2 starts from the transverse foramen of the sixth cervical vertebra to the transverse foramen of the second cervical vertebra; V3 starts from where it leaves the transverse foramen of the second cervical vertebra through the foramen magnum, piercing dura matter; and V4 starts from the posterior intracranial fossa. Bilateral V4 segments unite in a prepontine cistern, forming a basilar artery. Anomalies of the VA are rare and are mostly incidentally diagnosed on cerebral imaging, as there are no clinical symptoms or signs as described in the literature. They are crucial to be diagnosed prior to performing any diagnostic radiology, interventional radiology, or carotid surgeries in the neck, particularly intravascular pathologies. The most common anomalies are presented on the left VA in approximately 2.4-5.8% of all cases and less predominantly on the right VA, occurring in 0.25%. Atypical vertebral arteries, double arteries, duplicated arteries, fenestrated arteries, tortuous arteries, elongated arteries, kinked arteries, aneurysmal arteries, and collagen diseases could possibly precipitate in strokes. Subclavian steal steno-occlusive disease can be diagnosed in some cases and is associated with reversing VA blood flow [3].

Aberrant anatomical origin of the VA is a very rare variant of the VA pathologies implicating not only cerebrovascular neurological events but also VA dissection and surgical anatomical exposure of crucial nearby structures, particularly in carotid artery interventions. The "vertebral arteria lusoria," although it is rarely seen, should be considered in patients undergoing esophageal surgeries, and unawareness of such pathology may cause life-threatening incidents. Moreover, dual origins of vertebral arteries should be noticed in the preoperative assessment of patients with extracranial vascular pathologies. Posterior cerebral circulation neurological events, vertebrobasilar insufficiency, or spinal cord ischemia are well-documented complications of VA injury in patients with normal or aberrant VA origins. The VA provides perfusion to the spinal cord. Therefore, the presence of the following factors-coverage of the artery of Adamkiewicz originating from T8 to L1, abdominal aortic open repair with a ligated inferior mesenteric artery, and an occluded iliac artery-are considered indications for preserving an aberrant VA. As part of the assessment of patients for elective procedures where ligation of the atypical VA is considered crucial, reperfusion ought to be approached whenever convenient. Surgical repair options for VA revascularization in case of ligation or scarification during the procedure are the same techniques performed with significant VA stenosis associated with neurological deficits. Options for the proximal part of the VA revascularization could be done by attachment of a coral patch to the aortic repair graft, vertebral-carotid transposition, vein interposition graft, or synthetic graft bypass. These techniques are associated with 0.9% of cerebrovascular events and death [4].

The patient presented in this case had vague symptoms of intermittent dizziness for over a week, which disappeared after carotid endarterectomy, raising suspicion that she was suffering from posterior circulation cerebral insufficiency due to the aberrant origin of the right VA above the area of stenotic ICA. She also had right hemispheric stroke symptoms that lasted for a few days but reversed to her baseline. Patients with posterior circulation symptoms tend to be treated medically as they are not attributed to carotid disease. This case demonstrates the importance of investigating patients with ischemic stroke with a cross-sectional angiogram at the time or after CT head to rule out carotid and vertebral disease as the cause of the cerebrovascular event but also to exclude such rare anatomy. A duplex scan is unlikely to discover such aberrant anatomy, as the origin of the VA was well above the common carotid artery bifurcation and beyond the angle of the mandible.

The literature review revealed only three published reports of a VA originating from the ICA, with only one reporting the aberrant origin of the right VA from the right ICA without an association with an aberrant subclavian artery or dual supply [5-7].

Each of these three case reports presented a single patient. The mean age of the patients was 68 years old. They all presented with symptoms suggestive of an ischemic cerebral stroke and included symptoms of paresthesia, dizziness, ataxia, and slurred speech. They shared dyslipidemia as a common co-morbidity. Each patient was diagnosed incidentally with an aberrant VA anatomy originating from the ICA on imaging. The three of them had different methods of diagnosis. The methods were ultrasound, CTA, and magnetic resonance angiography. They were all found to have stenosis in the ipsilateral ICA, but each had a different percentage of stenosis. The mean was 83.3% stenosis of the ipsilateral ICA. Two of the three patients were treated, and both underwent surgical treatments. One was treated similarly to our case with a carotid endarterectomy and a patch repair, but they did a vertebral endarterectomy simultaneously as well. The other patient was treated with carotid artery stenting. In all three cases, the post-operative period was uneventful. In addition, they all showed patent ICA and VA on post-operative follow-up ultrasound after an average of eight months [5-7].

## Conclusions

In cases with carotid artery stenosis presenting with posterior circulation symptoms, it is vital to thoroughly investigate patients with a CTA in addition to a CT head to rule out the aberrant anatomy of the VA before deciding to treat (or manage medically). In this case, a standard carotid endarterectomy with patch repair was required as the VA arose distal to the diseased ICA. Awareness of the atypical vertebral arteries, which are mostly not associated with any symptoms, is crucial for the vascular surgeon when planning surgery in order to minimize the risk of iatrogenic injuries and stroke in the perioperative period. Being familiar with atypical VA reperfusion techniques is fundamental in specific case scenarios in carotid endarterectomy surgeries, as they could need to be done.
